# Immunosuppressive role of CD11b^+^CD33^+^HLA‐DR^−^ myeloid‐derived suppressor cells‐like blast subpopulation in acute myeloid leukemia

**DOI:** 10.1002/cam4.3360

**Published:** 2020-08-11

**Authors:** Shin Young Hyun, Eun Jung Na, Ji Eun Jang, Haerim Chung, Soo Jeong Kim, Jin Seok Kim, Jee Hyun Kong, Kwang Yong Shim, Jong In Lee, Yoo Hong Min, June‐Won Cheong

**Affiliations:** ^1^ Department of Internal Medicine Yonsei University Wonju College of Medicine Kangwon‐do South Korea; ^2^ Avison Biomedical Research Center Yonsei University College of Medicine Seoul South Korea; ^3^ Department of Internal Medicine Yonsei University College of Medicine Seoul South Korea

**Keywords:** acute myeloid leukemia, arginase, inducible nitric oxide synthase, myeloid‐derived suppressor cells, prognosis

## Abstract

**Objective:**

Myeloid‐derived suppressor cells (MDSCs) facilitate tumor growth and development by suppressing T cell function; however, their role in acute myeloid leukemia (AML) remains unclear. Here, we investigated the immunosuppressive role and prognostic value of blasts with an MDSC‐like phenotype.

**Methods:**

CD11b^+^CD33^+^HLA‐DR^−^ MDSC‐like blasts from bone marrow mononuclear cells of patients with AML were analyzed. To investigate their T cell‐suppressing function, MDSC‐like blasts were isolated using flow cytometry and co‐cultured with CD8^+^ cytotoxic T cells and NB4 leukemic cells. Treatment outcomes were then compared between the MDSC‐like blasts low (≤9.76%) and high (>9.76%) groups to identify clinical significance.

**Results:**

MDSC‐like blasts showed higher expression of arginase‐1 and inducible nitric oxide synthase. Isolated MDSC‐like blasts significantly suppressed CD8^+^ T cell proliferation induced by phytohemagglutinin A. NB4 cell proliferation was significantly suppressed upon co‐culture with CD8^+^ cytotoxic T cells and partially restored upon co‐culture with MDSC‐like blasts. Patients with high MDSC‐like blasts at diagnosis showed substantially shorter overall survival and leukemia‐free survival relative to low MDSC‐like blasts patients, with subgroup analysis showing statistically significant differences in patients not receiving allogeneic hematopoietic stem cell transplantation.

**Conclusion:**

We demonstrated that MDSC‐like blasts drive AML‐specific immune‐escape mechanisms by suppressing T cell proliferation and restoring T cell‐suppressed NB4 cell proliferation, with clinically higher fractions of MDSC‐like blasts at diagnosis resulting in poor prognosis.

## Graphical abstract

CD11b^+^CD33^+^HLA‐DR^‐^MDSC‐like blasts play a role in immune‐escape mechanisms of AML, thereby facilitating proliferation of leukemic cells. This represents the first study offering evidence of CD11b^+^CD33^+^HLA‐DR^‐^MDSC‐like blasts as an adverse prognostic marker for AML.

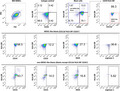

## 1 INTRODUCTION

Despite several new treatment strategies for acute myeloid leukemia (AML), such as the FMS‐like tyrosine kinase 3 inhibitors and the isocitrate dehydrogenase 1/2 inhibitors, have emerged, the treatment of AML remains a significant challenge as most of the responses are transient and resistance to drugs occurs.^1^ Cancer cells decrease the activity of various immune cells in order to prevent an immune response while also selectively inducing cells with immunosuppressive functions, such as inactive dendritic cells, regulatory T (Treg) cells, and tumor‐associated macrophages. Among these, myeloid‐derived suppressor cells (MDSCs) are derived from immature myeloid cells originating from hematopoietic stem cells (HSCs) and produced when differentiation of immature myeloid cells into mature myeloid cells ceases under specific conditions, such as cancer, various infectious diseases, trauma, bone marrow (BM) transplantation, and certain autoimmune disorders.^2^ MDSCs play an immunosuppressive role by suppressing T cell and natural killer cell responses in tumors. Additionally, MDSCs induce the production of Treg cells, also involved in immunosuppression, thereby facilitating tumor proliferation and distant metastasis.^3^ MDSCs express CD11b and CD33 as myeloid markers but not human leukocyte antigen complex‐DR isotype (HLA‐DR), and can be categorized into CD15^−^ monocytic MDSCs and CD15^+^ granulocytic MDSCs.^2^, ^4^, ^5^, ^6^

Numerous recent studies report the involvement of MDSC accumulation in tumor‐associated immunosuppression in various cancer types, which is a phenomenon shared by most cancer types.^7^, ^8^, ^9^, ^10^, ^11^ Additionally, elevated MDSC levels are associated with cancer progression, low rates of therapeutic response, and low survival rates.^12^, ^13^, ^14^, ^15^ In hematologic malignancies, several studies confirmed the presence of MDSCs and their immunosuppressive function promoting tumor proliferation; however, the precise phenotype and associated mechanism associated with this immunosuppression remain unknown. A recent study targeted multiple myeloma isolated mononuclear MDSCs (CD11b^+^CD14^+^HLA‐DR^−/low^) and polynuclear MDSCs (CD11b^+^CD14^−^CD33^+^CD15^+^) from the BM and demonstrated their immunosuppressive function in vitro.^16^, ^17^ Additionally, in B cell non‐Hodgkin's lymphoma, CD14^+^HLA‐DR^−^ MDSCs contribute to tumor progression,^18^ and in chronic myeloid leukemia, polynuclear MDSCs are present along with elevated arginase‐1 levels, with in vitro experiments revealing their role in suppressing T cell proliferation.^19^ However, few studies have examined the roles of MDSCs in AML and their significance as a prognostic factor.

Leukemic cells and MDSCs in AML originate from HSCs in the BM, with both generated by the early maturation arrest of immature myeloid cells during HSC development. Additionally, leukemic cells and MDSCs share the feature of arginase‐dependent immunosuppression.^20^, ^21^ Therefore, we hypothesized that MDSC‐specific immunosuppressive functions might play a specific role in the onset and progression of AML. This study examined whether CD11b^+^ CD33^+^HLA‐DR^−^ MDSC‐like blasts with the same phenotype as MDSC are present in the BM of AML patients and whether these cells exert immunosuppressive functions to promote leukemic cell proliferation. Additionally, we assessed the effects of these cells on the prognosis of AML.

## 2 MATERIALS AND METHODS

### 2.1 Primary leukemia cells and leukemia cell lines

Primary leukemia cells were isolated from the diagnostic BM aspiration of patients with de novo AML diagnosed at Yonsei University Severance Hospital between 2006 and 2017. Mononuclear cells (MNCs) were isolated by Ficoll‐Hypaque density gradient centrifugation and then cryopreserved. Specimens were collected according to a protocol approved by the Severance Hospital Institutional Review Board for clinical sample procurement, and informed consent was obtained in accordance with the Declaration of Helsinki.

RS4;11, Molm13, MV4‐11, and NB4 human leukemia cell lines (American Type Culture Collection) were maintained in Roswell Park Memorial Institute‐1640 medium supplemented with 10% (v/v) heat‐inactivated fetal bovine serum, 100 U/mL penicillin, and 10 µg/mL streptomycin in a 5% CO_2_ humidified incubator at 37°C.

### 2.2 MDSC isolation and flow cytometric analysis

To isolate MDSC‐like blasts, fresh BM MNCs at diagnosis were isolated, and single‐cell suspensions (2 × 10^6^ cells) were washed with phosphate‐buffered saline containing 2% fetal calf serum, followed by labeling with anti‐CD33‐FITC (BD Biosciences), an anti‐HLA‐DR‐peridinin chlorophyll protein complex (BD Biosciences), and anti‐CD11b‐phycoerythrin (BD Biosciences) for 30 minutes at room temperature. These cells were rewashed with cold Dulbecco's phosphate‐buffered saline at 4°C and resuspended with 5 µL of phycoerythrin‐labeled goat isotype‐matched mouse IgG antibody (BD Biosciences), which served as the control. Then, CD11b^+^CD33^+^ HLA‐DR^−^ blasts were sorted using a BD FACSAria (BD Biosciences) cell sorter. After isolation, expression of CD14, CD15, induced nitric oxide synthase (iNOS), and arginase‐1 (ARG1) on CD11b^+^CD33^+^HLA‐DR^−^ blasts was assessed with the following respective antibodies: anti‐CD33‐PE, anti‐HLA‐DR‐APC, anti‐CD11b‐Pacific blue (Beckman Coulter), anti‐CD14‐FITC, anti‐CD15‐FITC, (BD Biosciences), anti‐iNOS (Santa Cruz Biotechnology), and anti‐ARG1 (R&D Systems). Prior to incubation with unconjugated iNOS and arginase antibodies, cells were fixed with 4% paraformaldehyde and permeabilized with permeabilizing buffer (Thermo Fisher). Conjugation with secondary FITC antibody was performed after incubation with primary anti‐iNOS or anti‐ARG1.

To measure the percentage of MDSC‐like blasts in all patients, CD11b^+^CD33^+^HLA‐DR^−^ blasts in BM were gated after selecting blasts by gating the MNC area based on forward scatter, side scatter, and CD45 positivity. Anti‐CD33‐PE, anti‐HLA‐DR‐Pacific blue, anti‐CD11b‐FITC, and anti‐CD45‐APC (Beckman) were used in this experiment. Flow cytometric analysis was performed using an LSR II flow cytometer and analyzed with Flow Jo software (BD Biosciences).

### 2.3 Cell culture and treatment conditions

To identify MDSC‐like blast‐mediated suppression of T cell proliferation, MDSC‐like blasts were isolated from BM MNCs of patients with AML and co‐cultured with CD8^+^ T cells from a healthy donor. The cells were treated with phytohemagglutinin A (PHA; 10 µmol/L; Sigma‐Aldrich) to induce CD8^+^ T cell proliferation, and after 1, 3, and 7 days, cell viability was determined to assess changes in PHA‐induced CD8^+^ T cell proliferation in the presence or absence of MDSC‐like blasts. We assessed the proliferation of CD8^+^ T cells using carboxyfluorescein diacetate succinimidyl ester (CFSE) staining and flow cytometry in this experiment.

To verify the ability of MDSC‐like blasts to facilitate leukemic cell proliferation, MNCs from the BM of three AML patients with high proportions of MDSC‐like blasts were used. Cryopreserved human peripheral blood CD8^+^ cytotoxic T cells were purchased from iXCells biotechnologies. Co‐cultures were set up with leukemic cells (RS4;11, Molm13, MV4‐11, or NB4), CD8^+^ T cells, and BM MNCs (from patients with high proportions of MDSC‐like blasts) seeded in transwells at a 4:1:1 ratio, respectively. The bottom well was initially seeded with BM MNCs, prior to seeding the upper layer with the leukemic cells and CD8^+^ T cells. Cells were maintained in RPMI‐1640 medium supplemented with 10% (v/v) heat‐inactivated fetal bovine serum, 100 U/mL penicillin, and 10 µg/mL streptomycin in a 5% CO_2_ humidified incubator at 37°C. Cell viability assays were used to evaluate patterns of changes of leukemic cell proliferation according to culture time (0, 24, and 72 hours).

### 2.4 Cell viability assay

The Cell Counting Kit‐8 assay (DOJINDO Molecular Technologies, Inc) was used to assess the change of proliferation of leukemic cells. The cells were cultured at the indicated conditions, followed by the addition of 10  µL of CCK‐8 solution to each well according to the manufacturer's protocol. The optical density was read at 450 nm using a microplate reader (VersaMax; Molecular Devices).

### 2.5 Patients and treatments

Among adult patients diagnosed with de novo AML at Severance Hospital from January 2006 through December 2017, 58 were enrolled in this study. Exclusion criteria included the following; patients with acute promyelocytic leukemia, incomplete clinical and laboratory data, including cytogenetics, those not receiving systemic remission induction chemotherapy, or unavailable BM MNC sample at the time of diagnosis. The diagnosis was made according to the consensus guidelines for the immunologic diagnosis of acute leukemia and the World Health Organization classification of Tumours of Haematopoietic and Lymphoid Tissues.^22^

Various clinical and biological characteristics at diagnosis, including age, sex, white blood cell count, platelet count, hemoglobin level, a fraction of blasts in peripheral blood (PB) and BM, French–American–British morphology, cytogenetics, molecular abnormalities, and immunophenotype, were retrospectively analyzed. Patients were divided into three prognostic groups based on cytogenetics and molecular abnormalities, as previously described.^23^ For remission induction, patients received chemotherapy comprising a 7‐day continuous infusion of standard dose cytarabine at 100 mg/m^2^ and idarubicin at 12 mg/m^2^ for 3 days (7‐3 protocol). Patients who achieved complete remission (CR) after one or two cycles of induction chemotherapy received additional postremission treatment. Allogeneic hematopoietic stem cell transplantation (HSCT) as postremission treatment was performed in 20 patients.

### 2.6 Statistical analysis

All experimental results are represented as the mean ± standard error of the mean (SEM). Each experiment was performed for at least three times. The exact sample size for each experiment is given in the figure legends. The paired *t* test and Student's *t* test were used for statistical analysis using GraphPad Prism version 7.00 for Windows (GraphPad Software, Inc). Differences with a *P < *.05 were considered statistically significant.

Various clinical and laboratory parameters, treatment responses, and survival data were compared between the two groups. CR was defined by normalization of blood counts and BM morphology and the disappearance of all signs of leukemia lasting for ≥ 4 weeks according to the recommendations of the International Working Group of the diagnosis and response criteria in AML.^24^ Leukemia‐free survival (LFS) was measured from the date of attainment of CR to the date of relapse at last follow‐up visit or death without relapse. Overall survival (OS) was measured from the time of diagnosis to the time of death from any cause. Follow‐up for survival analysis was performed up to 5 years after diagnosis. The distributions of continuous variables in different patient groups were compared using Student's *t* test, and binary variables were compared using the Chi‐squared test, the Fisher's exact test, and Pearson's Chi‐squared test. Survival curves were calculated using the Kaplan‐Meier method and analyzed using the log‐rank test. Differences with a *P < *.05 were considered statistically significant, and all statistical calculations were performed with PASW in SPSS (v.25.0; IBM Corp.).

## 3 RESULTS

### 3.1 MDSCs are present in the BM of AML patients with various proportions

**FIGURE 1:**
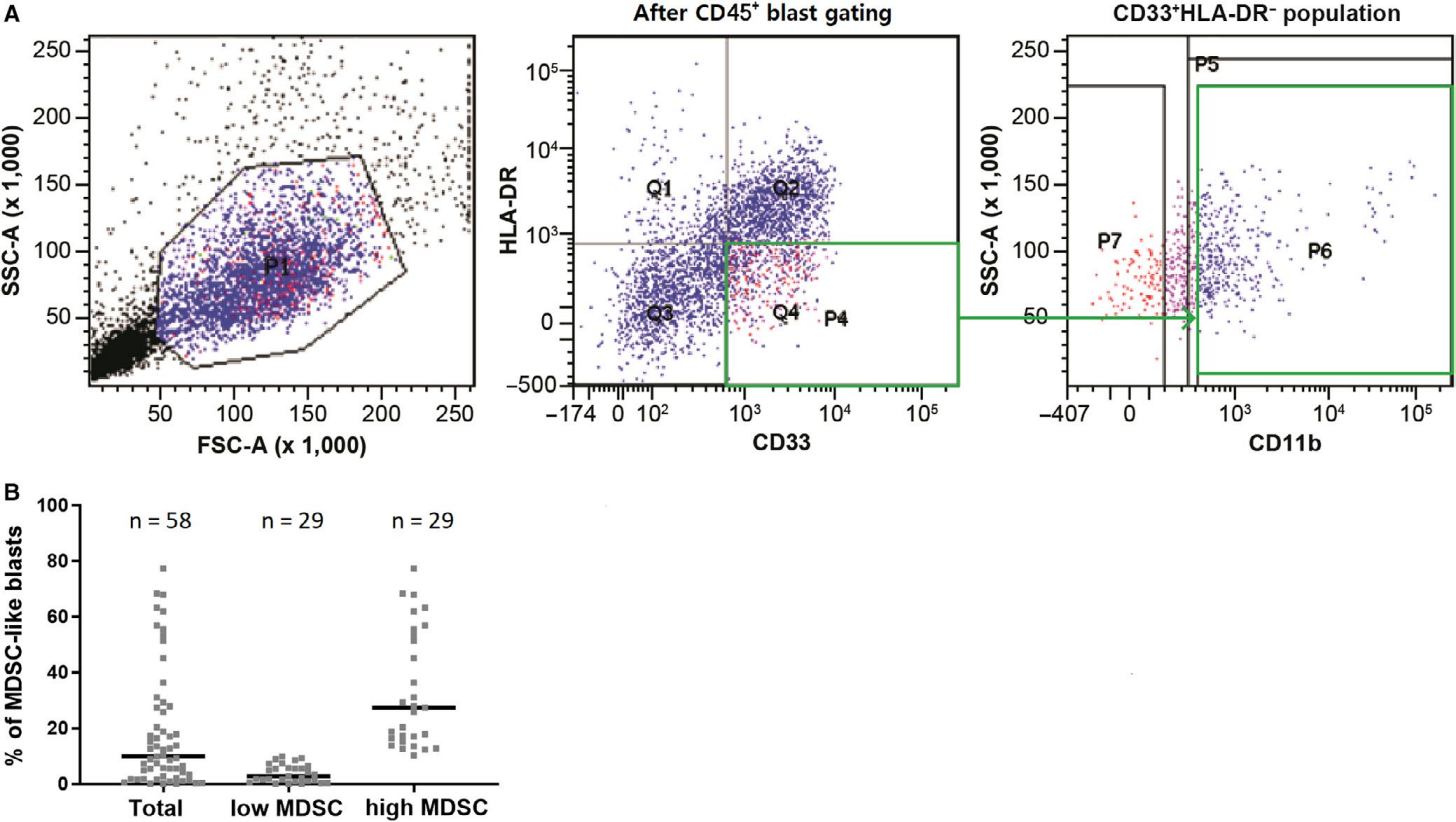
CD11b^+^CD33^+^HLA‐DR^−^ MDSC‐like blasts identified in bone marrow aspirate from patients with de novo acute myeloid leukemia. A, Multicolor flow cytometry staining CD33, HLA‐DR, and CD11b which determine the existence of the CD11b^+^CD33^+^HLA‐DR^−^ MDSC‐like blasts subpopulation within leukemic blasts. B, Frequency distribution of CD11b^+^CD33^+^HLA‐DR^−^ MDSC‐like blasts in the bone marrow. A total of 58 patients were classified into two groups (low or high) according to the frequency of MDSC‐like blasts. Bars represent median values. Abbreviations: MDSC, myeloid‐derived suppressor cells; Low MDSC, patients with low MDSC‐like blasts (bone marrow MDSC‐like blasts ≤ 9.76%); High MDSC, patients with high MDSC‐like blasts (bone marrow MDSC‐like blasts > 9.76%)

We first determined whether the BM of AML patients comprised MDSC‐like blasts. Among BM blasts from 58 AML patients, we quantified the levels of MDSC‐like blasts by flow cytometry using a gating strategy presented in Figure 1A. According to forward scatter/side scatter/CD45 positivity among the MNC population, blasts were gated by CD11b^+^CD33^+^HLA^−^DR^−^. The proportion of MDSC‐like blasts out of all BM blasts was 18.11 ± 2.474% (mean), with a median of 9.76% (0.02‐77.37) (Figure 1B).

**FIGURE 2:**
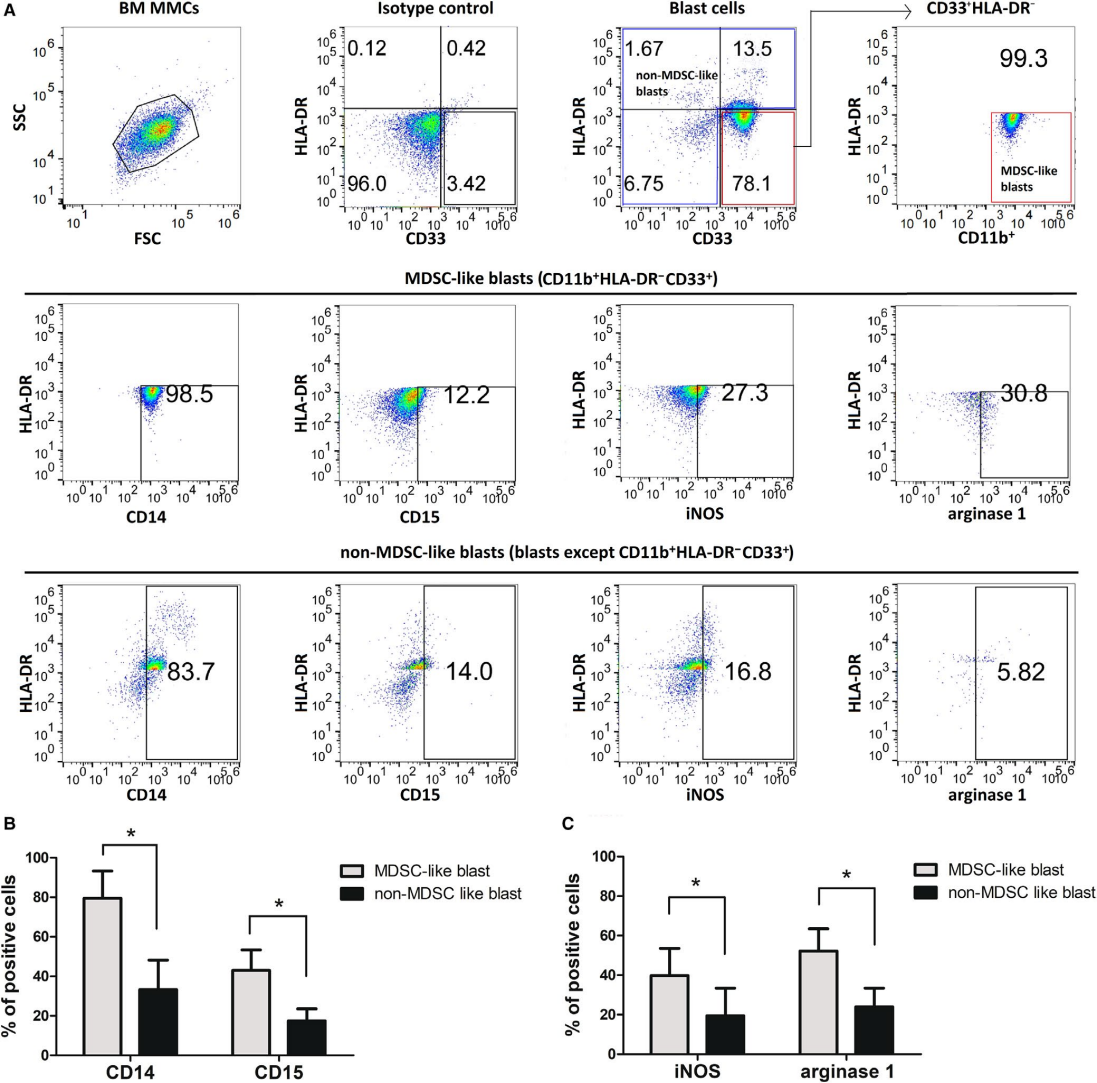
Flow cytometric analysis of CD14, CD15, induced nitric oxide synthase, and arginase‐1 in CD11b^+^CD33^+^HLA‐DR^−^ MDSC‐like blasts. A, Representative figure of flow cytometric analysis of CD14, CD15, induced nitric oxide synthase, and arginase‐1 in BM MDSC‐like blasts. B, BM MDSC‐like blasts from patients with AML showed higher CD14 and CD15 expression relative to other blasts from the same patients (n = 6). C, Bone marrow MDSC‐like blasts from patients with AML showed higher induced nitric oxide synthase and arginase‐1 expression relative to other blasts from the same patients (n = 6). Abbreviations: AML, acute myeloid leukemia; BM, bone marrow; MNCs, mononuclear cells; MDSC, myeloid‐derived suppressor cells

MDSC‐like blasts included monocytic and granulocytic subpopulation. Although blasts excluding MDSC‐like blasts produced negligent levels of CD14 and CD15 expression, the proportion of MDSC‐like blasts expressing CD14 was 79.52% and expressing CD15 was 43.01% (Figure 2A). These results indicated that MDSC‐like blasts among AML blasts mainly comprised monocytic subtype, with subsets of granulocytic subtype.

### 3.2 MDSC‐like blasts from AML patients significantly suppress iNOS and ARG1 expression, as well as T cell proliferation

Then we verified whether MDSC‐like blasts from AML patients were capable of suppressing T cell function as similar as MDSC. The percentage of cells expressing iNOS was 39.75 ± 13.78% for MDSC‐like blasts, and 19.44 ± 14.03% for the other blasts than MDSC‐like blasts (*P = *.03), and that for ARG1 was 52.17 ± 11.31% for MDSC‐like blasts and 23.92 ± 9.503% for other blasts than MDSC‐like blasts (*P = *.01) (Figure 2B). This finding that most MDSC‐like blasts expressed iNOS and ARG1 at higher levels relative to the other blasts implies that these MDSC‐like blasts might exert immunosuppressive functions via increased iNOS and ARG1 levels.

**FIGURE 3:**
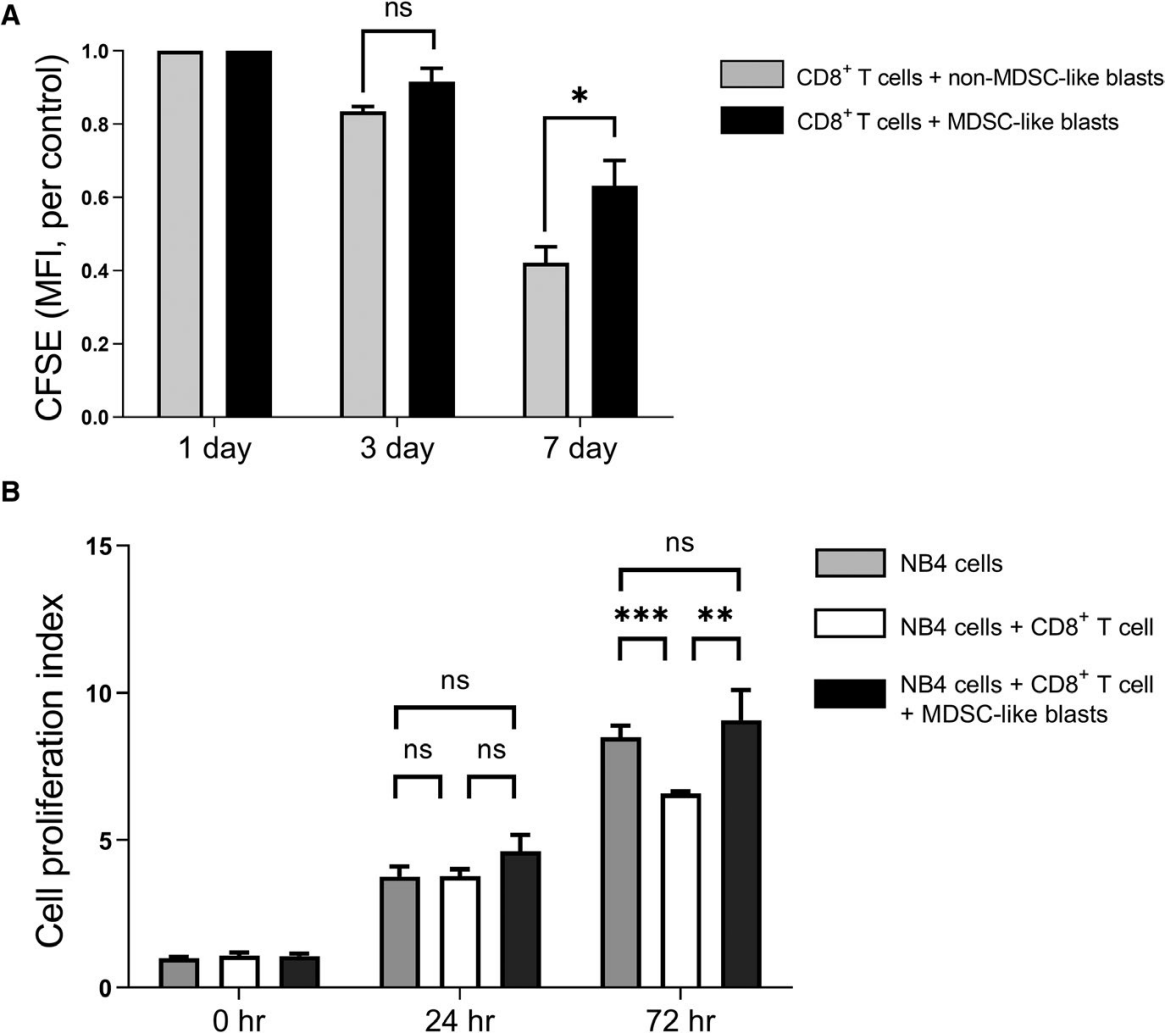
The immunosuppressive function of CD11b^+^CD33^+^HLA‐DR^−^ MDSC‐like blasts from the BM of acute myeloid leukemia patients. A, Phytohemagglutinin A‐induced CD8^+^ T cell proliferation was significantly suppressed during co‐culture with MDSC‐like blasts as compared with co‐culture with other blasts (n = 9). B, Suppressed proliferation of NB4 leukemia cells by CD8^+^ cells was partially restored by co‐cultured MDSC‐like blasts enriched BM mononuclear cells (n = 9). ^*^ *P* < .05, ^**^ *P* < .01, ^***^ *P* < .001, ^****^ *P* < .0001. Abbreviations: BM, bone marrow; CFSE, carboxyfluorescein diacetate succinimidyl ester; MDSC, myeloid‐derived suppressor cells; MFI, mean fluorescence intensity

MDSC‐like blasts were then cultured with CD8^+^ T cells at a 1:1 ratio in order to determine the altered levels of PHA‐induced T cell proliferation. Compared with CD8^+^ T cells co‐cultured with other blasts, those co‐cultured with MDSC‐like blasts showed a decreased level of PHA‐induced proliferation, which was seen through a small decrease in CFSE staining over 72 hours (Figure 3A). This confirmed the ability of MDSC‐like blasts to more strongly suppress T cell proliferation than other blasts.

### 3.3 MDSC‐like blasts partially restore the proliferation of suppressed NB4 leukemia cells by CD8^+^ T cells

Next, we verified whether MDSC‐like blasts affect leukemic cell proliferation. Due to the varying proportions of MDSC‐like blasts among patients, we used the cryopreserved BM MNCs from AML patients who showed a high percentage of MDSC‐like blasts for this experiment. The proportion of MDSC‐like blasts in three patients was 44.61%, 39.17%, and 23.00%, respectively. In co‐cultures of NB4 cells and CD8^+^ T cells without MDSC‐like blast enriched MNCs, NB4 cell proliferation was decreased by CD8^+^ T cells (*P* = .0061), whereas this suppressed proliferation of NB4 cells by CD8^+^ T cells was partially restored in co‐cultures of NB4 cells and CD8^+^ T cells with MDSC‐like blast enriched MNCs (*P* = .0343) (Figure 3B).

### 3.4 The proportion of MDSC‐like blasts in BM significantly influences therapeutic outcomes

Patients were divided into high and low groups based on the median value of MDSC‐like blasts proportion among AML blasts (9.76%). Patient characteristics for both groups are shown in Table 1. The median (range) fraction of MDSC‐like blast in the high group (n = 29) was 27.37% (10.04‐77.37%), and that in the low group (n = 29) was 1.77% (0.01‐9.76%) (Figure 1B). There were no significant differences between groups in terms of age, sex, white blood cell count, hemoglobin level, platelet count, lactate dehydrogenase level, and percentage of BM blasts. However, the proportion of patients with poor cytogenetic risk was higher in the high group than in the low group (*P = *.013). Further, there was a difference in the proportion of MDSC‐like blasts among the risk groups; the median percentages of MDSC‐like blasts in the favorable, intermediate, and reduced risk groups were 6.35%, 17.56%, and 30.85%, respectively (*P = *.009).

**TABLE 1:** Comparison of pretreatment patient characteristics in CD11b^+^CD33^+^HLA‐DR^−^ MDSC‐like blast in low and high groups

Variables	Low group	High group	*P*‐value
Number of patients	29	29	
Age (y)	45.0 ± 14.4	46.4 ± 17.7	.740
Male (%)	18 (62.1%)	20 (69.0%)	.058
WBC count (×10^6^/L)	16 417 ± 21 718	36 748 ± 47 471	.043
Hemoglobin (g/dL),	8.6 ± 2.8	8.6 ± 2.6	.936
Platelet count (×10^6^/L),	56 ± 42	64 ± 53	.494
Lactate dehydrogenase (IU/L),	511 ± 439	659 ± 468	.226
Blasts in BM (%)	54.2 ± 25.5	54.5 ± 23.6	.087
Favorable/intermediate/poor molecular/cytogenetic risk groups (n)	9/17/3	3/14/12	.013

Note

Continuous variables were presented as mean ± SD.

Abbreviations: BM, bone marrow; High group, patients with high MDSC‐like blasts (BM MDSC‐like blasts > 9.76%); Low group, patients with low MDSC‐like blasts (BM MDSC‐like blasts ≤ 9.76%); MDSC, myeloid‐derived suppressor cells; SD, standard deviation;WBC, white blood cell.

**FIGURE 4:**
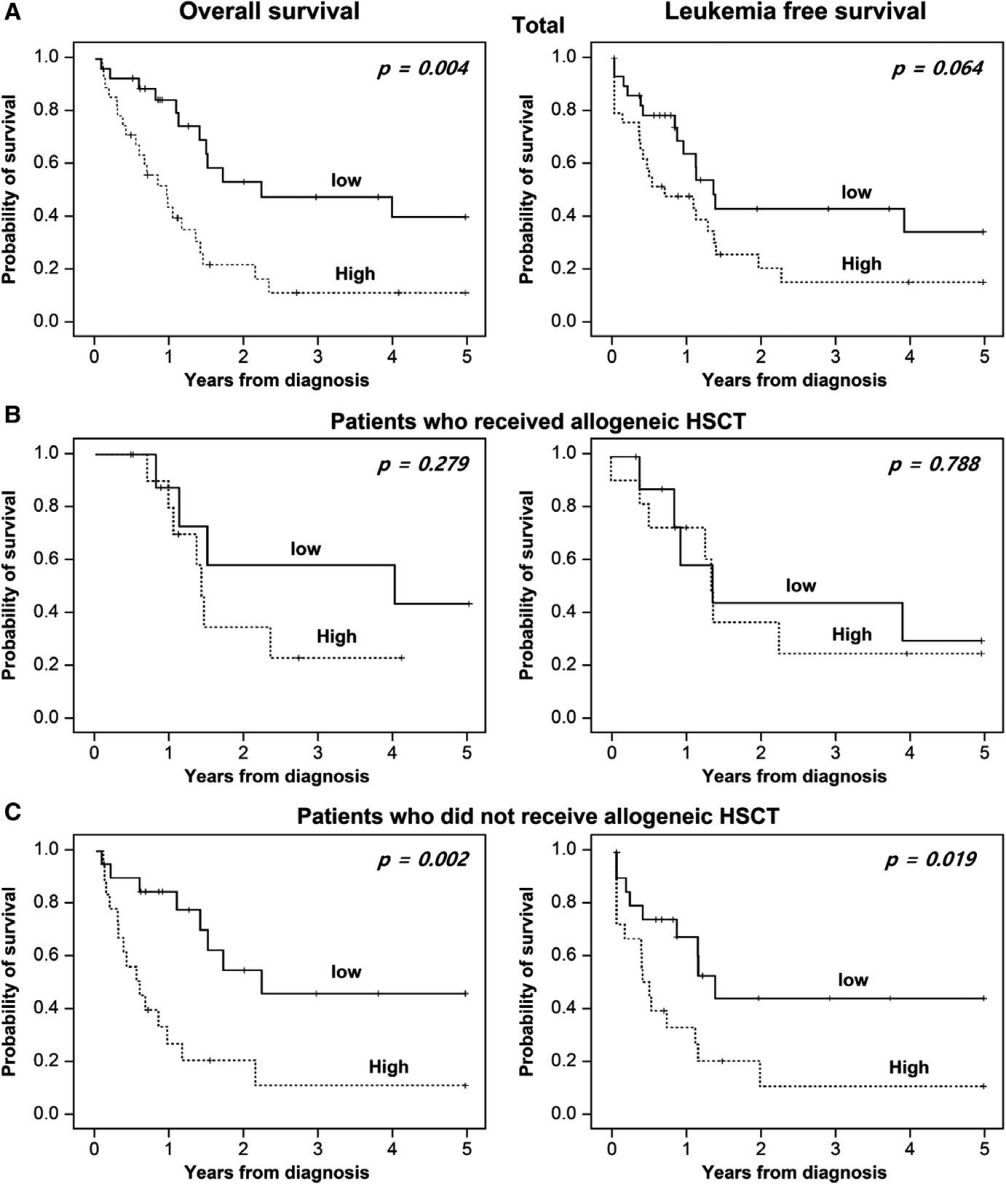
Overall survival and leukemia‐free survival rates in the high and low MDSC‐like blast groups. A, Total patients (n = 58), B, patients receiving allogeneic hematopoietic stem cell transplantation (n = 29), and C, patients not receiving hematopoietic stem cell transplantation (n = 29). Abbreviations: High, patients with high MDSC‐like blasts (bone marrow MDSC‐like blasts > 9.76%); Low, patients with low MDSC‐like blasts (bone marrow MDSC‐like blasts ≤ 9.76%)

Among all patients, CR was achieved in 49 of 58 patients (85%), with the CR rate to induction chemotherapy in the high group not significantly different from that in the low group (Table 2). However, patients in the high group displayed a significantly shorter OS rate than patients in the low group (*P = *.004), as well as a lower LFS rate relative to patients in the low group (Table 2, Figure 4A). Notably, in subgroup analysis, a patient who did not receive allogeneic HSCT showed significant differences in OS and LFS between high and low groups (Figure 4B), whereas patients who received allogeneic HSCT did not show any difference between groups (Figure 4C).

**TABLE 2:** Treatment outcome of 58 patients receiving remission‐induction therapy in the low and high MDSC‐like blast groups

Variables	Low group	High group	*P*‐value
Number of patients	29	29	
CR (%)	26 (89.7%)	23 (79.3%)	.277
Allogeneic HSCT in first CR (%)	9 (31.0%)	11 (37.9%)	.581
Deceased (%)	13 (44.8%)	22 (75.9%)	.024
OS days, mean ± SD	1106 ± 148	557 ± 117	.004
LFS days, mean ± SD	910 ± 161	512 ± 123	0.064

Note

Continuous variables were presented as mean ± SD.

Abbreviations: CR, complete remission; High group, patients with high MDSC‐like blasts (bone marrow MDSC‐like blasts > 9.76%); HSCT, hematopoietic stem cell transplantation; LFS, leukemia‐free survival; Low group, patients with low MDSC‐like blasts (bone marrow MDSC‐like blasts ≤ 9.76%); MDSC, myeloid‐derived suppressor cells; OS, overall survival; SD, standard deviation.

Univariate analysis of OS revealed that a higher percentage of MDSC‐like blasts (*P = *.005) and age >50 years (*P = *.038) were significantly related to a short OS. Multivariate analysis confirmed that a higher percentage of MDSC‐like blast represented a predictive factor for short OS (hazard ratio [HR]: 3.418; 95% confidence interval [CI]: 1.580‐7.394; *P = *.002) as well as LFS (HR: 2.022; 95% CI: 0.936‐4.366; *P = *.073) (Table 3). In the subgroup analysis, a higher percentage of MDSC‐like blasts showed a strong potential for predicting OS (HR: 3.062; 95% CI: 1.258‐7.452; *P = *.014) and LFS (HR: 2.126; 95% CI: 0.889‐5.086; *P = *.090) in patients who did not receive allogeneic HSCT, but it did not show potential as a prognostic factor in patients who received allogeneic HSCT (Table S1).

**TABLE 3:** Univariate and multivariate COX regression analyses of prognostic factors for overall survival and leukemia‐free survival in AML patients

Covariates	Overall survival			Leukemia‐free survival		
	HR	95% CI	*P*‐value	HR	95% CI	*P*‐value
Univariate analysis						
%CD11b^+^CD33^+^HLA‐DR^−^> 9. 76%	2.759	1.354‐5.623	.005	1.850	0.945‐3.621	.072
Age > 50 y	2.046	1.037‐4.031	.038	1.565	0.812‐3.013	.181
Male gender	1.430	0.715‐2.861	.312	1.264	0.638‐2.503	.502
WBC count > 50 × 10^9^/L	1.177	0.508‐2.724	.704	1.339	0.582‐3.081	.492
Bone marrow blast > 50%	1.444	0.708‐2.945	.312	2.033	1.015‐4.074	.045
Cytogenetics/molecular risk: poor	1.410	0.673‐2.951	.362	1.520	0.747‐3.094	.248
Not receiving allogeneic HSCT	0.579	0.767‐3.249	.215	1.541	0.766‐3.100	.225
Multivariate analysis						
%CD11b^+^CD33^+^HLA‐DR^−^> 9.76%	3.418	1.580‐7.394	.002	2.022	0.936‐4.366	.073
Cytogenetics/molecular risk: poor	1.224	0.533‐2.810	.634	0.984	0.429‐2.270	.970
Not receiving allogeneic HSCT	1.300	0.557‐3.033	.544	1.723	0.815‐3.642	.154
Age > 50 y	2.541	1.081‐5.983	.033			
Bone marrow blast > 50%				0.547	0.262‐1.139	.107

Abbreviations: AML, acute myeloid leukemia; CI, confidence interval; HR, hazard ratio; HSCT, hematopoietic stem cell transplantation; WBC, white blood cell.

## 4 DISCUSSION

Here, we demonstrated that CD11b^+^CD33^+^HLA^−^DR^−^ MDSC‐like blasts existed in varying proportions in BM from AML patients, and confirmed that these MDSC‐like blasts contribute to the proliferation of AML cells. To our knowledge, this is the first study that used MDSC‐like blast isolated from the BM of patients with AML for direct co‐culture. Unlike the other blast population in BM from the same patients, MDSC‐like blasts showed elevated iNOS and ARG1 levels, implying their involvement in the ability of MDSC to suppress T cell‐specific immune function in AML. Co‐culture of MDSC‐like blasts with CD8^+^ T cells and NB4 leukemic cells revealed that NB4 cell proliferation suppressed by CD8^+^ T cells was partially restored in the presence of MDSC‐like blasts, thereby suggesting an immunosuppressive role of MDSC‐like blasts. As expected, these MDSC‐like blasts influenced prognosis, indicating lower survival rates in patients with high proportions of MDSC‐like blasts and especially in patients not receiving allogeneic HSCT. This is the first study that suggested MDSC‐like blast level in BM at the time of diagnosis as a prognostic factor in AML.

Previous studies consistently show that MDSCs in PB contribute to the progression of various cancers by reducing T cell response via expression of iNOS, ARG1, and inflammatory cytokines.^2^ MDSCs are immature myeloid cells that can be categorized as granulocytic (CD11b^+^CD33^+^CD14^−^HLA‐DR^−^) and monocytic (CD14^+^HLA‐DR^−^)^5^ MDSCs. Most studies of MDSCs in hematologic malignancies have been targeting lymphoid tumors, such as lymphoma and multiple myeloma, and showed that increased levels of granulocytic and monocytic MDSCs exist in lymphoid neoplasms.^16^, ^25^, ^26^, ^27^, ^28^ Azzaoui et al detected MDSCs in the PB of patients with diffuse large B cell lymphoma and showed that increased CD14^+^ monocytic MDSC levels suppressed T cell proliferation, and that depletion of these cells restored this activity.^25^ Additionally, they showed a correlation between the immature granulocyte fraction and the expression of ARG1 and indoleamine‐pyrrole 2,3‐dioxygenase, suggesting the production of reactive oxygen species and Treg cells rather than ARG1 as the immunosuppressive mechanisms associated with monocytic MDSCs. By contrast, in the present study, more than half of BM MDSC‐like blasts from AML patients expressed CD14 as well as ARG1, possibly suggesting that monocytic MDSC‐like blasts suppress T cell function via ARG1 in AML. Recently, Francis M et al demonstrated an arginase‐dependent ability of AML blasts to suppress the T cell proliferation of alloreactive T cells stimulated by allogeneic dendritic cells, as well as the differentiation of murine granulocyte‐monocyte and human CD34^+^ progenitors. In addition, the immunosuppressive activity of AML blasts can be modulated through an arginase inhibitor.^21^ This result described an arginase‐dependent immune suppressive effect of AML blast that is consistent with our results. However, our study suggested that the MDSC‐like blast subpopulation is present in AML blasts at various proportions and that this subpopulation serves a T cell‐suppressive function. High proportions of this MDSC‐like blast subpopulation may be one of the multiple factors contributing to poor prognosis.

The present study demonstrated the existence of blast subpopulation resembling the phenotype and immunosuppressive role of MDSCs in AML. Only two studies have focused on MDSCs in AML, but none of them found an MDSC‐like blast population in the BM of AML patients. A recent study by Pyzer et al defined CD11b^+^HLA‐DR^−^CD14^∓^CD33^+^CD15^−^ and CD11b^+^HLA‐DR^−^CD14^−^CD33^−^CD15^+^ as monocytic and granulocytic MDSCs, respectively, among PB MNCs from AML patients and according to flow cytometry.^29^ Their results showed an average of 7.9% MDSCs present in the PB of AML patients (vs 0.7% in the peripheral blood of a healthy individual), and that co‐culture of an AML cell line with PB monocytes from a healthy individual resulted in expansion of MDSCs, suggesting that MDSC levels increase during AML onset and progression. Moreover, Sun et al quantified CD33^+^CD11b^+^HLA‐DR^−^ cells in BM from AML patients, revealing elevated levels of CD33^+^CD11b^+^HLA‐DR^−^ monocytic MDSCs.^26^ The limited number of studies concerning MDSCs in AML might be due to the difficulty associated with MDSC isolation, given that PB and BM contain mixtures of immature myeloid cells, leukemic cells, and MDSCs according to the characteristics of the specific condition. BM of AML harbors various immature myeloid cells and leukemic cells, with the latter exhibiting high heterogeneity, even within a single patient.^30^ Furthermore, both MDSCs and leukemic cells belong to the category of immature myeloid cells; therefore, flow cytometry considers the two cell types as an overlapping entity with no marked difference between forward and side scatters. In the case of MDSCs in mouse models, a clear marker, such as Gr1^−^, for defining MDSCs is available; however, in humans, no standard cellular phenotype exists to distinguish between MDSCs and leukemic cells. The two previously cited studies used a method of excluding leukemic cells based on their known phenotype; however, the possibility that a proportion of leukemic cells was included in the analysis cannot be eliminated. To differentiate the two cell types, flow cytometric analysis using a broad range of color combinations is required. Nevertheless, it remains challenging to isolate and analyze cells during an experiment using multiple patient samples.^31^ The study by Pyzer et al raised similar concerns, and although they used cytogenetic abnormalities to distinguish clones, no uniform identity was capable of completely distinguishing between MDSCs and malignant clones.^29^ Furthermore, specific MDSCs express cytogenetic abnormalities also displayed by AML cells; therefore, the possibility that the MDSCs might have originated from the AML subclone could not be eliminated. Based on these findings, we hypothesized that blasts with the same phenotype as MDSC are present and exert immunosuppressive functions like MDSCs, and the assumption is confirmed by this experiment.

One strength of this study is its suggestion of MDSC‐like blasts as a poor prognostic factor, especially in AML patients who did not receive allogeneic HSCT. This might be attributed to intensive chemotherapy and immunotherapy based on HSCT completely removing the minimal residual disease, including residual MDSC‐like blasts, thereby successfully suppressing their function and overcoming the associated poor prognosis associated with their elevated levels. A role for MDSCs as a prognostic factor was recently suggested for hematologic malignancies, such as Hodgkin's lymphoma, multiple myeloma, acute leukemia, and chronic myeloid leukemia.^19^, ^26^, ^27^, ^32^, ^33^, ^34^, ^35^ Sun et al identified elevated MDSC levels in the BM of patients with a high level of minimal residual disease relative to those with low levels, as well as decreased MDSC levels in the BM upon CR after chemotherapy for AML.^26^ Moreover, Azzaoui et al confirmed a correlation between increased monocytic MDSC levels in PB and prognostic index, event‐free survival, and circulating Treg cells in patients with diffuse large B cell lymphoma.^25^ In AML, as in other hematologic malignancies, MDSCs appear to affect prognosis adversely. Future studies are needed to determine whether allogeneic HSCT can help overcome MDSC‐related adverse prognostic effects.

This study has several limitations. First, it is unclear whether both MDSC‐like blasts and the rest of blasts are originated from same leukemic clone or not. To confirm this, it may be helpful to conduct comparative analysis based on flow cytometry, molecular genetic analysis, and functional assays on MDSC‐like blast and other blasts. The second limitation is that it was difficult to divide the MDSC‐like blasts and other blasts into two populations that are completely separated by immunophenotype. As already discussed, flow cytometry results showed no marked difference in forward scatter and side scatter between MDSC‐like blasts and other blasts, and there are no known immunophenotypic markers till date for clearly differentiating between MDSC and leukemic blasts. Finally, we could not demonstrate statistical significance in the multivariate analysis for LFS. Because a total of nine patients (three in low MDSC group and six in high MDSC group) who did not achieve CR were not included in the multivariate analysis for LFS, the sample size consequently became too small (n = 49) to elicit true statistical significance. So, additional studies having a larger sample size will be needed.

In conclusion, this study verified the presence of CD11b^+^CD33^+^HLA^−^DR^−^ blasts which resembled MDSC in the BM of newly diagnosed AML patients and demonstrated their ability to suppress the immune functions of T cells by increasing iNOS and ARG1 expression. Additionally, the presence of MDSC‐like blasts adversely affected the prognosis of AML, indicating that allogeneic HSCT might help overcome such adverse effects. Further research is needed to clarify the association between MDSC‐like blasts and specific molecular pathways in AML to find novel treatment modalities that can overcome the adverse prognostic effects of MDSC‐like blasts.

## CONFLICT OF INTEREST

The authors have no conflict of interests.

## AUTHOR CONTRIBUTION

SY Hyun designed the study, contributed essential materials and facilities, analyzed the data, and wrote the paper; J.‐W. Cheong designed the research, contributed essential materials and facilities, analyzed the data, and wrote the paper; YH Min designed the research, and contributed essential materials and facilities; EJ Na performed the experiments, analyzed, and interpreted the data; JE Jang, HR Cheong, SJ Kim, JS Kim, JH Kong, KY Shim, and JI Lee contributed essential materials and facilities.

## Supporting information

Table S1

## Data Availability

The data that support the findings of this study are available from the corresponding author upon reasonable request.
