# Supplementary material for: Immunosuppressive role of CD11b+CD33+HLA‐DR− myeloid‐derived suppressor cells‐like blast subpopulation in acute myeloid leukemia
Source: Cancer Med. 2020 Aug 11;9(19):7007–17. doi: 10.1002/cam4.3360 (PMC7541151; doi:10.1002/cam4.3360)
Supplement: Supplementary file 1 — Table S1 [file CAM4-9-7007-s001.docx]

**Supplementary table**

| **TABLE 1** Univariate and multivariate COX regression analyses of prognostic factors for overall survival and leukemia-free survival in AML patients who did not receive allogeneic HSCT | | | | | | |
| --- | --- | --- | --- | --- | --- | --- |
| **Covariates** | **Overall survival** | | | **Leukemia-free survival** | | |
|  | **HR** | **95% C.I.** | ***P*-value** | **HR** | **95% C.I.** | ***P*-value** |
| *Univariate analysis* |  |  |  |  |  |  |
| %CD11b^+^CD33^+^HLA-DR^-^ > 9. 76% | 3.174 | 1.358-7.418 | 0.008 | 2.266 | 1.011-5.079 | 0.047 |
| Age > 50 years | 2.302 | 0.913-5.806 | 0.077 | 1.803 | 0.752-4.326 | 0.187 |
| Male gender | 1.714 | 0.742-3.960 | 0.208 | 0.629 | 0.275-1.440 | 0.272 |
| WBC count > 50 x 10^9^/L | 0.859 | 0.320-2.303 | 0.762 | 0.777 | 0.291-2.074 | 0.614 |
| Bone marrow blast > 50% | 0.589 | 0.264-1.316 | 0.197 | 0.505 | 0.227-1.120 | 0.093 |
| Cytogenetics/molecular risk: poor | 1.689 | 0.694-4.109 | 0.248 | 1.712 | 0.710-4.127 | 0.231 |
| *Multivariate analysis* |  |  |  |  |  |  |
| %CD11b^+^CD33^+^HLA-DR^-^ > 9.76% | 3.062 | 1.258-7.452 | 0.014 | 2.126 | 0.889-5.086 | 0.090 |
| Cytogenetics/molecular risk: poor | 1.138 | 0.448-2.892 | 0.786 | 1.210 | 0.469-3.124 | 0.693 |
| Abbreviations: AML, acute myeloid leukemia; HR, hazard ratio, C.I. confidence interval; WBC, white blood cell; HSCT, hematopoietic stem cell transplantation | | | | | | |
